# Ectopic Overexpression of *SsCBF1*, a CRT/DRE-Binding Factor from the Nightshade Plant *Solanum lycopersicoides*, Confers Freezing and Salt Tolerance in Transgenic *Arabidopsis*

**DOI:** 10.1371/journal.pone.0061810

**Published:** 2013-06-03

**Authors:** Lili Zhang, Zhenjun Li, Jingfu Li, Aoxue Wang

**Affiliations:** 1 College of Life Sciences, Northeast Agricultural University, Harbin, China; 2 State Key Laboratory of Plant Genomics, National Centre for Plant Gene Research, Institute of Genetics and Developmental Biology, Chinese Academy of Sciences, Beijing, China; 3 College of Horticulture, Northeast Agricultural University, Harbin, China; 4 Heilongjiang Provincial Key University Laboratory of Agricultural Biological Functional Genes, Northeast Agricultural University, Harbin, China; Kyushu Institute of Technology, Japan

## Abstract

The C-repeat (CRT)/dehydration-responsive element (DRE) binding factor (CBF/DREB1) transcription factors play a key role in cold response. However, the detailed roles of many plant CBFs are far from fully understood. A CBF gene (*SsCBF1*) was isolated from the cold-hardy plant *Solanum lycopersicoides*. A subcellular localization study using GFP fusion protein indicated that SsCBF1 is localized in the nucleus. We delimited the SsCBF1 transcriptional activation domain to the C-terminal segment comprising amino acid residues 193–228 (SsCBF1^193–228^). The expression of *SsCBF1* could be dramatically induced by cold, drought and high salinity. Transactivation assays in tobacco leaves revealed that SsCBF1 could specifically bind to the CRT *cis*-elements in vivo to activate the expression of downstream reporter genes. The ectopic overexpression of *SsCBF1* conferred increased freezing and high-salinity tolerance and late flowering phenotype to transgenic *Arabidopsis*. RNA-sequencing data exhibited that a set of cold and salt stress responsive genes were up-regulated in transgenic *Arabidopsis*. Our results suggest that SsCBF1 behaves as a typical CBF to contribute to plant freezing tolerance. Increased resistance to high-salinity and late flowering phenotype derived from SsCBF1 OE lines lend more credence to the hypothesis that plant CBFs participate in diverse physiological and biochemical processes related to adverse conditions.

## Introduction

During the long evolutionary history, plants have developed various strategies to cope with environmental stresses such as drought, high salinity and low temperature. Cold stress has adverse effects on growth and yield of many important crops and seriously restricts their cultivation and distribution [1], [2]. Plants can acclimate in response to cold stress through physiological and biochemical processes triggered by the induced expression or repression of a variety of genes [3].

An essential *cis*-acting element, C-repeat (CRT) or dehydration-responsive element (DRE), was commonly found in the promoter regions of many low-temperature and drought-responsive genes [4]–[7]. Two types of AP2 domain-containing transcription factors which specifically bind to the CRT/DRE element and activate the expression of downstream genes were isolated using yeast one-hybrid screening [5], [8]. The first type, called C-repeat Binding Factor/DRE Binding protein 1 (CBF/DREB1), could recognize the regulatory CRT/DRE element present in the promoters of many cold-inducible genes. However, the *CBF/DREB1* genes were proved to be induced by low temperature but not by dehydration and salt stresses [8], [9]. The second type of transcription factors, called DRE binding protein 2 (DREB2), are encoded by the genes that are induced by dehydration and salt stresses but not by low temperature stress [8], [10].

In *Arabidopsis*, there are three major CBF/DREB1 proteins which play a critical role in the regulation of many cold-stress related genes [3]. The presence of conserved signature sequences, PKK/RPAGRxKFxETRHP and DSAWR that bracket the AP2/EREBP DNA binding domains in the CBF proteins from various species, serves to distinguish them from other AP2/ERF family members [11]. Previous landmark studies have unveiled that the signature sequence of PKK/RPAGRxKFxETRHP found in CBF1 proteins not only represents the nuclear localization signal [12], but also contributes to the ability of the CBF1 proteins to bind to their DNA recognition sequence, the CRT/DRE element and therefore it is essential for the expression of CBF-targeted genes [13], [14] . Constitutive overexpression of the *CBF/DREB1* genes in *Arabidopsis* under control of the 35S promoter leads to enhanced freezing, drought and high-salinity tolerance [8], [15]–[17].

The feedback repression of the three CBFs in *Arabidopsis* was also previously proved to maintain the optimal regulation of cold-induced genes [18], [19]. Meanwhile, the transgenic plants described above also showed growth retardation phenotype under normal conditions. Bioinformatic analysis of microarray and RNA-seq data was used to identify the downstream genes of CBF/DREB1 [20]–[23] and help us better understand the cold-response mechanism [24]. These genes refer to various physiological processes and biochemical pathways. Because of the successful application of *Arabidopsis CBF/DREB1* genes in improving stress tolerance, the homologous genes have been cloned from many other plants such as rice, *Brassica napus*, barley, cherry and wheat [11], [25]–[30].

*Solanum lycopersicoides* (LA2408), collected at higher altitudes (up to 3600 meters) than any of other *Lycopersicon* species, is a wild nightshade distant-allied to cultivated tomato. Many traits of *S. lycopersicoides* including cold tolerance, resistance to virus diseases and insect pests were previously confirmed [31], [32]. Thus, it is an ideal candidate plant for isolating cold tolerance-related genes. In this study, we successfully cloned the full-length cDNA of the CBF1 from *S. lycopersicoides* which was designated as *SsCBF1* and performed the functional characterization based on phenotypic and bioinformatic analyses using transgenic approach. The ectopic overexpression of *SsCBF1* in *Arabidopsis* resulted in enhanced plant tolerance to freezing and salt stress. The goal of our research is to get a deep insight into the functional behaviors of the plant CBFs and illustrate the possibility that SsCBF1 may mediate responses to a wider range of environmental stresses other than cold stress.

## Materials and Methods

### Plant material, growth conditions and stress treatments

Seeds of *S. lycopersicoides* (LA2408) were kindly provided by TGRC (http://tgrc.ucdavis.edu), USA. Seeds of cultivated tomato cv. Castlemart (CM), *N. benthamiana* and *Arabidopsis thaliana* ecotype Col-0 were obtained from Tomato institute, Northeast Agricultural University (Harbin, China). Seedlings of *S. lycopersicoides* and CM were grown in a growth chamber maintained under 16 h of light (150 μE m^–2^ s^–1^) at 28°C and 8 h of dark at 18°C. *N. benthamiana* was grown under the same conditions.

*Arabidopsis thaliana* ecotype Col-0 was used as the wild-type. *35S_pro_*:*SsCBF1* T_3_ homozygous transgenic plants were obtained from transformation of Col-0 plants with the corresponding construct and used for all subsequent assays. *Arabidopsis* seeds were surface sterilized for 15 min in 10% bleach, washed five times with sterile water, and plated on half-strength Murashige and Skoog (MS) medium containing 0.8% (w/v) Bacto Agar [33]. Sterilized seeds were stratified at 4°C for 2 d in darkness and then transferred to a climate chamber set at 22°C with a 16-h-light/8-h-dark photoperiod. All experiments were repeated at least three times.

Abiotic stress treatments were applied 8 h after the switch to the light phase and plant material was exposed to continuous illumination (150 μE m^–2^ s^–1^) for the entire treatment period. For low-temperature stress, detached leaves of *S. lycopersicoides* seedlings were placed on two layers of filter paper soaked with water (0.02% Tween-20) and then transferred to a growth chamber set at 4°C. For salt treatment, detached leaves were placed on two layers of filter paper soaked with NaCl solution (250 mM, 0.02% Tween-20) or water (0.02% Tween-20) as a control. Drought stress was performed by placing detached leaves on two layers of dry filter paper. Leaves of four-week-old seedlings were used for all the above stress treatments. Samples were collected at the indicated time points and immediately frozen in liquid nitrogen. Plant material was stored at −80°C prior to RNA extraction.

### Gene isolation and analysis of the deduced amino acid sequence

Total RNA was extracted from leaves of *S. lycopersicoides* seedlings using the TRIzol® Plus RNA Purification Kit (Ambion, Austin, TX, USA). Degenerate PCR was performed using degenerate primers: D–F (5′- CCGAARAAGCCAGCTGGCAG -3′) and D–R (5′-AGCGGCCGCCTTTTGAATATC-3′). Flanking sequences of *SsCBF1* were retrieved using the rapid amplification of cDNA ends (RACE) method (Takara, Japan). Bioedit, DNAMAN and MEGA4.0 software were used to perform bioinformatics analysis. Southern Blotting analysis was performed as described by Xiao *et al* [34]. Genomic DNA of *S. lycopersicoides* was digested with three restriction enzymes *Bam*H I, *Hin*d III and *Xba* I, whose recognition sites were absent in the sequence of *SsCBF1*. The *SsCBF1* coding region probe was prepared by North & South® Chemiluminescent Hybridization and Detection Kit (PIERCE, IL, USA).

### Subcellular localization of SsCBF1

The *SsCBF1*-*eGFP* fusion gene was prepared for the subcellular localization experiment. Full length *eGFP* was PCR-amplified from pEGFP-C1 plasmid DNA using primers of *eGFP*-*Ss* fusion primer-F and *BstE*II-*eGFP* primer-R. *SsCBF1* was PCR amplified from the reverse transcription product with primers *Nco*I-*Ss* primer-F and *Ss-eGFP* fusion primer-R. The *Ss*-*eGFP* fusion gene was obtained by SOE-PCR (Splicing by Overlap Extension PCR). Primers used in this section were listed in Table S1. Finally, *Nco* I+*Bst*E II-digested *Ss*-*eGFP* fusion gene was inserted into the *Nco* I+*Bst*E II-digested pCAMBIA1302 vector to generate *35S_pro_*:*SsCBF1-eGFP*. The new recombinant vector was introduced into the *Agrobacterium* strain LBA4404 by a freeze-thaw method [35]. *N. benthamiana* leaves were used for the transient expression of *SsCBF1-eGFP* fusion gene. Fluorescence was detected with a Laser Scanning Confocal Microscope (NiKon A1R/A1, Japan).

### Preparation of transactivation constructs

*35S_pro_*:*SsCBF1* was used as one of the effector constructs. *AtCBF1* was PCR amplified and ligated into the *Bam*H I and *Sac* I restriction sites of plant expression vector pBI121 to generate the other effector construct *35S_pro_*:*AtCBF1*. The *COR15A* promoter was PCR-amplified with primers F: 5′-CACCCTTCGGAACAACAACAAGAGTTATTATGC-3′ and R: 5′-AGACCTATTACACTCCAAAATTACACG-3′ and cloned into pENTR using the pENTR Directional TOPO cloning kit (Invitrogen, USA). To generate *COR15A* promoter with mutations, site-directed mutagenesis was used to delete the three CRT elements using TaKaRa MutanBEST kit. Pairs of primers: Pro_mu_-F1, Pro_mu_ -R1, Pro_mu_ -F2, Pro_mu_ -R2, Pro_mu_ -F3 and Pro_mu_ –R3 were listed in Table S1. Then, both *COR15A* promoter versions were fused with the *eGFP* reporter gene and cloned into plant binary vector pBI121 to generate the reporter constructs *COR15A_pro_*:*eGFP* and *COR15A-m_pro_*:*eGFP*. The LUC reporter assays were performed as described by [36], [37]. The min35S and 4×CRTmin35S promoter sequences were PCR amplified and cloned into pCAMBIA1381Z which was previously modified by our lab members to generate the reporter constructs *min35S_pro_*:*LUC* and *4×CRTmin35S_pro_*:*LUC*. *Agrobacterium*-mediated infiltration of *N. benthamiana* leaves was performed as described [34], [38]. *N. benthamiana* leaves infiltrated with corresponding constructs were incubated at 28°C for 72 h before CCD imaging. A low-light cooled CCD imaging apparatus (NightOWL II LB983 with indigo software) was used to capture the LUC image and to count luminescence intensity. The leaves were sprayed with 100 mM luciferin and were placed in dark for 5 min before detecting luminescence. Five independent determinations were assessed.

### SsCBF1 fragments in the yeast pGBKT7 vector

To analyze the C-terminal activation domain of SsCBF1, we used the pGBKT7 vector and the yeast strain Y2H Gold with *His* and *Ade* as Gal4-dependent reporter genes. The full-length and derivatives of *SsCBF1* were PCR-amplified and cloned into the *Bam*H I/*Eco*R I sites of the pGBKT7 vector. These constructs were used to map the transcriptional activation domain of SsCBF1 in yeast. Amino acid replacement (W224A) within the conserved motif LWNYS was performed by PCR-mediated mutagenesis and amplification. Yeast two-hybrid assays were performed according to the instructions of the Matchmaker Gold Yeast Two-Hybrid System (Clontech). The transformed yeasts were suspended in liquid SD/-Trp to OD  = 0.5 and diluted to the indicated concentrations. Five microliters of suspended yeast cells was dropped on the SD plates and incubated at 30°C. The presence of transactivation activity was confirmed by yeast growth on the SD/-Trp/-His/-Ade plate. X-a-Gal (4 mg/ml) was used for the detection of α-galactosidase. Primers used are listed in Table S1.

### Quantitative RT-PCR analysis

Total RNA was extracted from plant materials with Trizol (Invitrogen, USA). Poly (dT) cDNA was prepared from 2 μg of total RNA with M-MLV reverse transcriptase (Promega, USA) and quantified with a thermocycler (Bio-Rad) with the SYBR *Premix Ex Taq* (Takara, Japan) according to the manufacturer's instructions. PCR was performed in 96-well optical reaction plates heated for 30 s at 95°C to activate hot start Taq DNA polymerase, followed by 45 cycles of denaturation for 5 s at 95°C, annealing for 30 s at 58°C, and extension for 30 s at 72°C. Expression levels of target genes were normalized to those of *ACTIN2*, *ACTIN1* and *ACTIN7* for *S. lycopersicoides*, tobacco and *Arabidopsis*, respectively [39]–[41]. Primers used to quantify gene expression levels are listed in Table S1. For semi-quantitative RT-PCR assay, RNA extraction and reverse transcription reaction were performed as mentioned above. The PCR conditions for amplification of *SsCBF1* were as follows: 5 min at 94°C, followed by 32 cycles of 15 s at 94°C, 30 s at 60°C, 20 s at 72°C. The same conditions were used in the amplification of *ACTIN7* of *Arabidopsis*, except that the number of PCR cycles was decreased to 20.

### Transformation of *Arabidopsis* and phenotypic analysis

*Agrobacterium* strain LBA4404 harboring the construct *35S_pro_*:*SsCBF1* was used for transformation of Col-0 by floral dip method [42]. Homozygous transformants of the T_3_ generation were selected for all of the following analyses.

Freezing tolerance assays for transgenic *Arabidopsis* were performed using a climate chamber. Seven-day-old transgenic and Col-0 plants grown on MS medium were transferred into the chamber set at −7°C for 8 h and thawed at 4°C for 12 h in the dark, and then returned to the standard growth conditions. About 50 plants of each genotype were used. Photographs were taken 3 days after treatment.

For seed germination investigation, 100 seeds of homozygous transgenic lines and Col-0 were placed on MS agar medium supplemented with none or NaCl of different concentrations for the stress treatment. Percentage of germinated seeds was recorded daily. Germination was defined as a clear sign of the emergence of radicle tip and the germination results were calculated based on four independent experiments. Photographs of the 150 mM NaCl treatment were taken 3 days after stratification.

For the root length assay, seeds from Col-0 and T_3_ transgenic plants were germinated on MS agar medium and grown vertically for 4 days, followed by transfer to fresh medium supplemented with or without 150 mM NaCl for vertical growth. The root length was measured with a ruler 11 days later and photographed.

The high-salinity resistance assay was performed using 3-week-old Col-0 and transgenic plants grown under standard conditions and irrigated with 300 mM NaCl solution (1 liter) every 3 days [43] until symptoms appeared (3 weeks).

### RNA-Seq analysis of transgenic *Arabidopsis*

RNA-seq analysis was carried out using one transgenic line (#11). Total RNA was isolated with Trizol reagent (Invitrogen, USA) from the aerial parts of the four-week-old seedlings of *35S_pro_*:*SsCBF1* and Col-0 plants grown in parallel under unstressed conditions. Materials from 20 plants of each genotype were pooled for RNA isolation. Sequencing was performed on the Illumina HiSeq 2000 platform. RNA-Seq results have been submitted to NCBI and can be accessed under the GEO accession number GSE40482. All changes in gene expression were statistically significant at Q-value <0.001 [44]–[47]. Pathways and Gene Ontology (GO) analysis were performed using Molecule Annotation System (MAS).

### Nomenclature

The electronic version of this article in Portable Document Format (PDF) in a work with an ISSN or ISBN will represent a published work according to the International Code of Nomenclature for algae, fungi, and plants, and hence the new names contained in the electronic publication of a PLOS ONE article are effectively published under that Code from the electronic edition alone, so there is no longer any need to provide printed copies.

In addition, new names contained in this work have been submitted to IPNI, from where they will be made available to the Global Names Index. The IPNI LSIDs can be resolved and the associated information viewed through any standard web browser by appending the LSID contained in this publication to the prefix http://ipni.org/. The online version of this work is archived and available from the following digital repositories: PubMed Central, LOCKSS.

## Results

### Comparison of cold resistance between *S. lycopersicoides* and cultivated tomato

**Figure 1:**
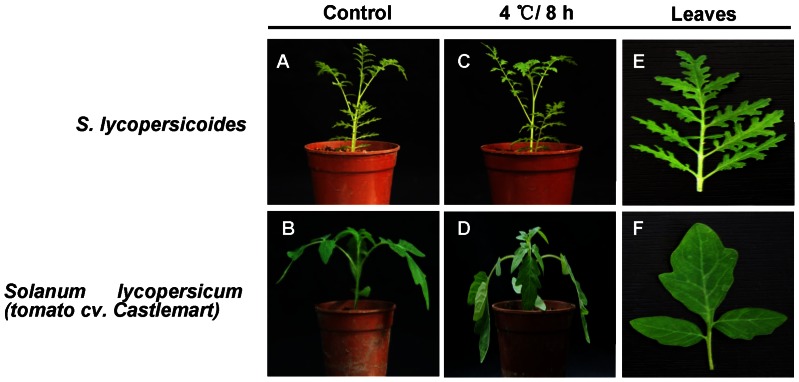
Low-temperature resistance test of *S. lycopersicoides* and CM. (**A–D**) Phenotypes of *S. lycopersicoides* and CM before (A, B) and after (C, D) 8 h of 4°C treatment. Seedlings of *S. lycopersicoides* and *Solanum lycopersicum* (tomato cv. Castlemart) *were g*rown under standard conditions and transferred to a climate chamber for the low-temperature stress. (**E–F**) Close-up shots of *S. lycopersicoides* and *Solanum lycopersicum* (tomato cv. Castlemart) leaves.

*S. lycopersicoide* has been collected at higher altitudes (up to 3600 meters) than any of the *Lycopersicon* species, a habitat subjected to chilling, dry and arid areas [48]. *S. lycopersicoide* (Fig. 1A) is a kind of dwarf shrub with serrate leaf margins (Fig. 1E). The leaf margins of cultivated tomato (*Solanum lycopersicum*) cv. Castlemart (CM) (Fig. 1B) are relatively smooth (Fig. 1F).

In order to preliminarily compare the cold-resistance of *S. lycopersicoides* and cultivated tomato cv. Castlemart (CM), four-week-old seedlings of the two species were exposed to 4°C for 8 h under continuous light (150 μE m^–2^ s^–1^). The result showed that *S. lycopersicoides* still kept vigorous and strong without any obvious signs of injury after 8 h of low-temperature stress (Fig. 1C). However, CM exhibited severe wilt symptoms and was relatively hypersensitive to cold stress (Fig. 1D).

### Isolation and sequence analysis of *SsCBF1*

**Figure 2:**
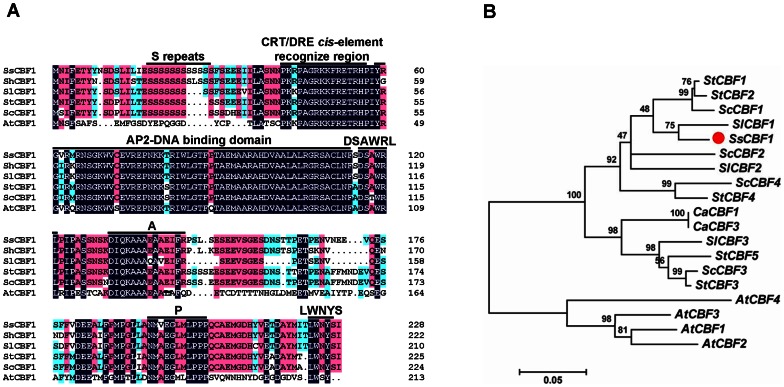
Sequence analysis of SsCBF1. (**A**) Amino acid sequence alignment between SsCBF1 and other known CBF1s. The alignment was performed using ClustalX 2.0 and DNAMAN software. Black background indicated conserved residues among all the proteins selected. The AP2 DNA-binding domain and other signature motifs were indicated by solid lines. (**B**) Phylogenetic relationships between SsCBF1 and other CBFs from various species. The phylogenetic tree was generated by the neighbor-joining method using MEGA 5.0. Organisms were abbreviated as follows: St, *Solanum tuberosum*; Sc, *Solanum commersonii*; Sl, *Solanum lycopersicum*; Ss, *Solanum lycopersicoides*; Ca, *Capsicum annuum*; At, *Arabidopsis thaliana*. GenBank accession numbers of the CBFs are listed as follows: AtCBF1 (AEE85066), AtCBF2 (AEE85064), AtCBF3 (AEE85065), AtCBF4 (ABV27186), SsCBF1 (ACY79412), SlCBF1 (AAS77820), SlCBF2 (AAS77821), SlCBF3 (AAS77819), ScCBF1 (ACB45093), ScCBF2 (ACB45094), ScCBF3 (ACB45092), ScCBF4 (ACB45084), StCBF1 (ABI74671), StCBF2 (ABI94367), StCBF3 (ACB45095), StCBF4 (ACB45083), StCBF5 (ACB45082), CaCBF1 (AAZ22480), CaCBF3 (ADM73296).

A cDNA fragment was isolated from *S. lycopersicoide* using the degenerate primers which were designed based on the conserved regions among other *Solanum* CBF1 proteins. Subsequently, the corresponding full-length cDNA sequence containing 51 bp of 5′ UTR, 138 bp of 3′ UTR and 687 bp of open reading frame (ORF) was cloned using RACE technology and designated as *SsCBF1* (Accession No. GU129700, NCBI). The *SsCBF1* gene encodes a putative protein of 228 amino acids with a predicted molecular mass of 25.4 kDa and isoelectric point (pI) of 4.85. Clustalx2.0 and DNAMAN were employed for generating sequence alignment among CBF1 proteins from different species. The result revealed that they shared highly conserved sequences in the AP2-DNA binding domain and CRT/DRE *cis*-element recognition region [13], [49]–[51]. Extended regions of high sequence identity were observed throughout the *Solanum* CBF1s. However, *Solanum* CBF1s shared relatively low homology with AtCBF1 beyond the above two highly conserved regions (Fig. 2A). Phylogenetic analysis based on the full-length protein sequences of CBF1s indicated that SsCBF1 is most closely related to SlCBF1 (Fig. 2B).

### The *S. lycopersicoides* CBF gene family contains at least five members

**Figure 3:**
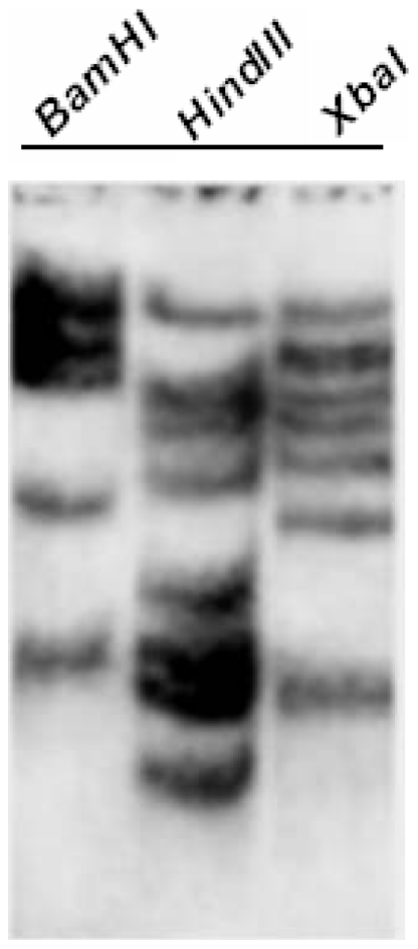
Southern blot analysis of the *SsCBF1* gene. *S. lycopersicoides* genomic DNA was digested with restriction enzymes *Bam*H I, *Hin*d III, and *Xba* I. The hybridization was performed using the full-length *SsCBF1* as a probe labeled with Digoxin.

To study the gene copy number of *SsCBF* in *S. lycopersicoides* genome, total DNA was digested with *Bam*H I, *Hin*d III or *Xba* I, of which recognition sites were absent in the *SsCBF1* gene sequence. The full-length *SsCBF1* nucleotide sequence was used as the probe labeled with Digoxin. Southern blotting generated 5, 8, and 7 bands in the *Bam*H I, *Hin*d III and *Xba* I digests, respectively, suggesting that *SsCBF1* belongs to a small gene family in the *S. lycopersicoides* genome (Fig. 3). It is also tempting to speculate that *S. lycopersicoides* genome contains at least five *SsCBF1*-related genes.

### *Ss*CBF1 transcription factor is targeted to the nucleus of plant cells

**Figure 4:**
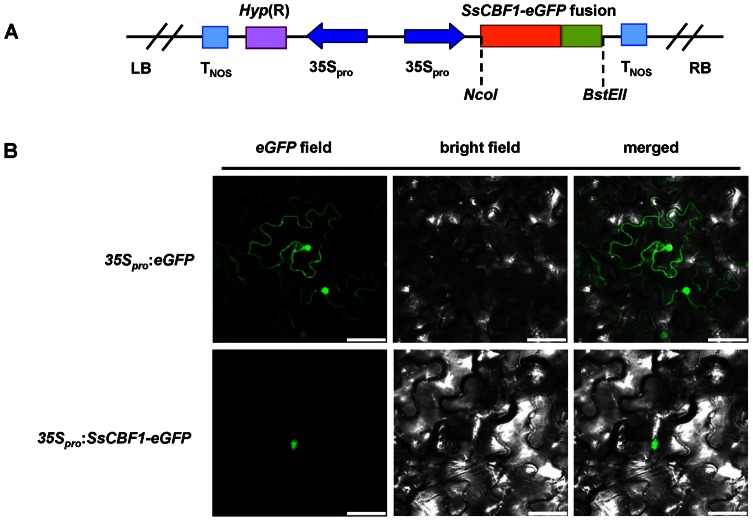
Subcellular localization of SsCBF1-eGFP fusion protein. (**A**) Schematic representation of *35S_pro_*:*SsCBF1*-*eGFP* construct used for transient expression in *N. benthamiana* leaves. (**B**) Subcellular localization assay of SsCBF1-eGFP fusion protein. *N. benthamiana* leaves transiently expressing eGFP alone (upper) and SsCBF1-eGFP (bottom) proteins were observed with a confocal microscope. Bars  = 50 µm.

A defining feature of transcription factors is to recognize and bind the *cis*-elements and then activate genomic gene expression in nucleus. To investigate the subcellular localization of the SsCBF1 protein, an *SsCBF1-eGFP* fusion gene was generated and ligated into pCAMBIA1302 under the control of CaMV35S promoter (Fig. 4A). An examination of the epidermal tissue of *N. benthamiana* leaves expressing fluorescent protein was accomplished by confocal laser scanning microscopy. The result showed that the SsCBF1-eGFP was observed solely in the nuclei, while the control eGFP signal (*35S_pro_*:*eGFP*) was detected in both nuclei and cytosol (Fig. 4B).

### SsCBF1 transactivate expression of *eGFP* driven by a CRT/DRE containing promoter

**Figure 5:**
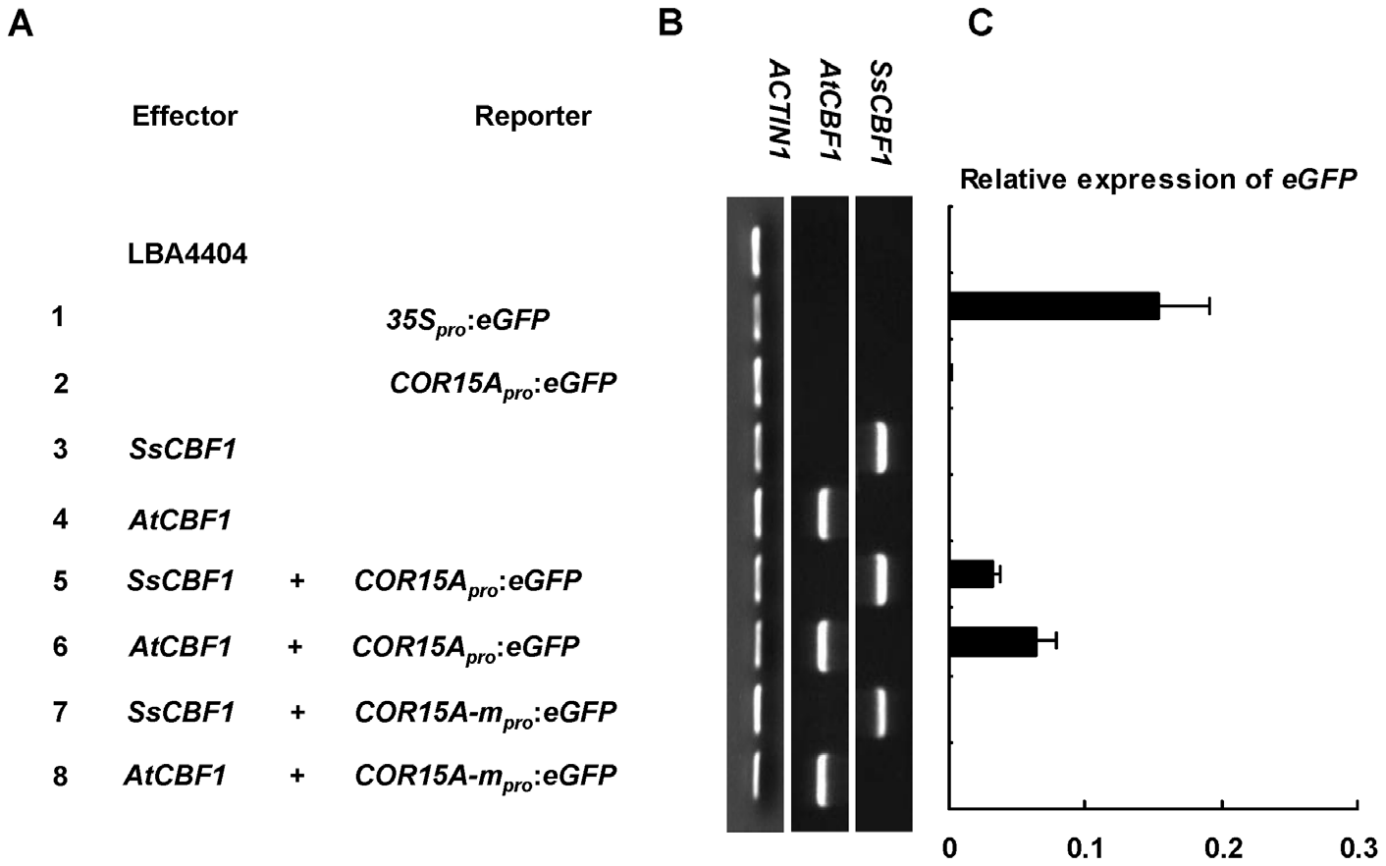
Transactivation of CRT/DRE *cis*-element containing promoter by SsCBF1 and AtCBF1. (**A**) Combinations of reporter and effector constructs used in the transient expression assays. *Agrobacterium* strain LBA4404 was used as a negative control. The *35S_pro_*:*eGFP* construct was used as a positive control. The reporter *eGFP* gene was driven by the wild-type or mutant *COR15A* promoter. The effectors *SsCBF1* and *AtCBF1* genes were driven by the CaMV35S promoter. (**B**) Semi-quantitative PCR detection of *SsCBF1*, *AtCBF1* and *ACTIN1* expression in tobacco leaves infiltrated with different combinations of constructs as shown in (A). (**C**) qRT-PCR analysis of *eGFP* expression. qRT-PCR procedure was as described in Methods. The results are representative of three independent experiments.

As a transcription factor, SsCBF1 should be tested for its transactivation activity. We expected that SsCBF1 could recognize the CRT/DRE *cis*-element in the promoters of target genes and activate their expression. Therefore, we designed two types of constructs [34], [38], one containing *AtCBF1* or *SsCBF1* gene as an effector driven by the CaMV35S promoter and the other containing *eGFP* gene as a reporter under the control of the cold-inducible COR15A promoter which includes three CRT/DRE elements [15]. *35S_pro_*:*eGFP* was used as a positive control. The mutant version of COR15A promoter was also introduced into this system. Both *Agrobacterium* strains harboring the effector and reporter construct were co-infiltrated into tobacco leaves (Fig. 5A). The *SsCBF1* and *AtCBF1* expression was confirmed by semi-RT-PCR using *ACTIN1* as an internal control (Fig. 5B). The *eGFP* expression was investigated by qRT-PCR to explore the interaction between SsCBF1 and CRT/DRE-containing promoter (Fig. 5C).

As shown in Figure 5C, high level of *eGFP* transcripts was detected with the *35S_pro_*:*eGFP* positive control construct (1). In addition, very low level of *eGFP* expression could be observed with the *COR15A_pro_*:*eGFP* construct only (2) (Fig. 5C). However, co-infiltration of *COR15A_pro_*:*eGFP* with a *35S_pro_*:*AtCBF1* or *35S_pro_*:*SsCBF1* effector construct led to an obvious induction of the *eGFP* expression (5,6) (Fig. 5C), suggesting that ectopic expression of SsCBF1 as well as AtCBF1 can activate *COR15A_pro_*:*eGFP* expression in this transient expression assay. In a parallel experiment, *COR15A-m_pro_*:*eGFP*, in which the three CRT/DRE elements of the *COR15A* promoter were deleted, together with *35S_pro_*:*AtCBF1* or *35S_pro_*:*SsCBF1* were co-infiltrated into tobacco leaves (7,8) (Fig. 5C). As shown in Figure 5C, the activation effects of SsCBF1 and AtCBF1 on *COR15A-m_pro_*:*eGFP* expression were both largely attenuated. These results revealed that SsCBF1 activates *COR15A* expression through direct association with the CRT/DRE *cis*-elements in *COR15A* promoter.

**Figure 6:**
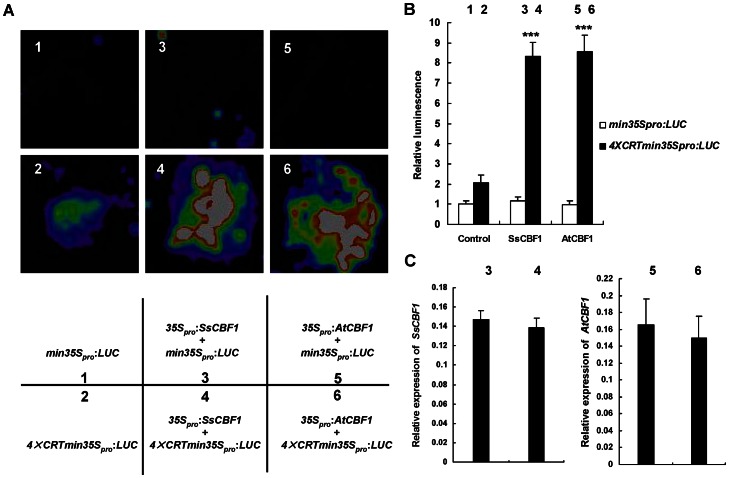
Transactivation of promoters with CRT elements only. (**A**) Transient expression assays showing that SsCBF1 and AtCBF1 specifically bind to the CRT elements and activate the expression of *LUC* reporter gene. The bottom panel indicates the combination of reporter and effector plasmids infiltrated. The *LUC* reporter gene is driven by a promoter with or without four tandem CRT elements fused upstream of a minimal (−46) 35S promoter sequence (*min35S_pro_*). The effector CBF1 genes are under the control of the CaMV35S promoter. Representative images of *N.benthamiana* leaves 72 h after infiltration are shown. (**B**) Quantitative analysis of luminescence intensity in (**A**). Five independent determinations were assessed. Error bars represent SD. Asterisks denote Student's *t*-test significance levels compared with the control: ***P<0.001. (**C**) qRT-PCR analysis of *SsCBF1* and *AtCBF1* expression in the infiltrated leaf areas shown in (**A**). Total RNAs were extracted from leaves of *N. benthamiana* coinfiltrated with the constructs. Five independent determinations were assessed. Error bars represent SD.

Further evidence supporting this CBF-CRT interaction came from another well-established transient expression assay in tobacco leaves. We verified the activation effect of SsCBF1 on the expression of firefly luciferase (*LUC*) reporter gene behind four copies of CRT *cis*-elements plus a minimal 35S promoter. Infiltration with *min35S_pro_*:*LUC* construct alone or the combination of *min35S_pro_*:*LUC* and *35S_pro_*:*AtCBF1* or *35S_pro_*:*SsCBF1*, the LUC activity could be barely detected (Fig. 6A, B). However, co-infiltration of *4×CRTmin35S_pro_*:*LUC* with the *35S_pro_*:*SsCBF1* or *35S_pro_*:*AtCBF1* resulted in strong luminescence intensity (Fig. 6A, 4B). The relatively low level of LUC signal observed with *4×CRTmin35S_pro_*:*LUC* construct alone could be due to the endogenous basal expression of CBF homologs. The above-described results suggest that SsCBF1 specifically bind to the CRT/DRE *cis*-elements to activate the expression of a gene in vivo.

### Fine-mapping of the putative transcriptional activation domain of SsCBF1

**Figure 7:**
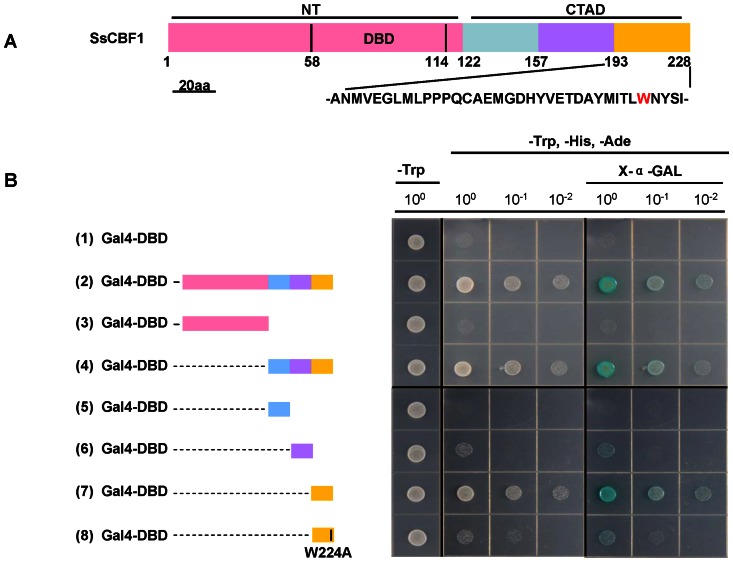
Functional dissection of the SsCBF1 C-terminal activation domain in yeast. (**A**) Schematic protein structure of SsCBF1. Full-length SsCBF1 and its derivatives (SsCBF1^0–121^, SsCBF1^122–156^, SsCBF1^157–192^, and SsCBF1^193–228^) were tested for the transactivation activity (see Methods for details). SsCBF1 derivatives were symbolized by filled color boxes. NT, N-terminal; CTAD, C-terminal activation domain; DBD, DNA-binding domain. Bar  = 20 amino acids (aa). (**B**) Mapping the putative transcriptional activation domain of SsCBF1 in yeast. Based on the schematic protein structure of SsCBF1, SsCBF1 and its derivatives including the mutant version of SsCBF1^193–228(W224A)^ were fused to the Gal4-DBD (pGBKT7). The empty pGBKT7 vector was used as a negative control. Survival of yeast cells on the selective media needs the presence of functional activator peptides. X-a-Gal was used to test the expression of α-galactosidase.

A typical plant CBF transcription factor contains two important domains: the N-terminal DNA-binding domain (DBD) and the C-terminal activation domain (CTAD) which mediated transcription initiation [13], [52]. Moreover, an increasing body of evidence proves that tryptophan residue (W) is a very important amino acid for the transcriptional activity of a transcription factor [53]–[55]. In our research, the SsCBF1 protein was divided into two parts, one containing N-terminal DBD and the other containing CTAD (Fig. 7A). The CTAD was subdivided into three fragments: SsCBF1^122–156^, SsCBF1^157–192^ and SsCBF1^193–228^ (Fig. 7A). Each fragment was ligated into pGBKT7 and introduced to Y2HGold yeast strain. As shown in Figure 7B, the activator function is exclusively due to the C-terminal segment comprising amino acid residues 193–228 (SsCBF1^193–228^) which harbors the LWNYS motif. In addition, the substitution of W by A (alanine) in the LWNYS motif severely abolished the transcriptional activity of this segment. Taken together, the amino acid residues 193–228 contribute to the activator potential of SsCBF1 and the tryptophan embedded in the LWNYS motif is a critical residue for the full transcriptional activity of SsCBF1.

### The *SsCBF1* transcripts accumulate in response to low temperature, drought and high salinity

**Figure 8:**
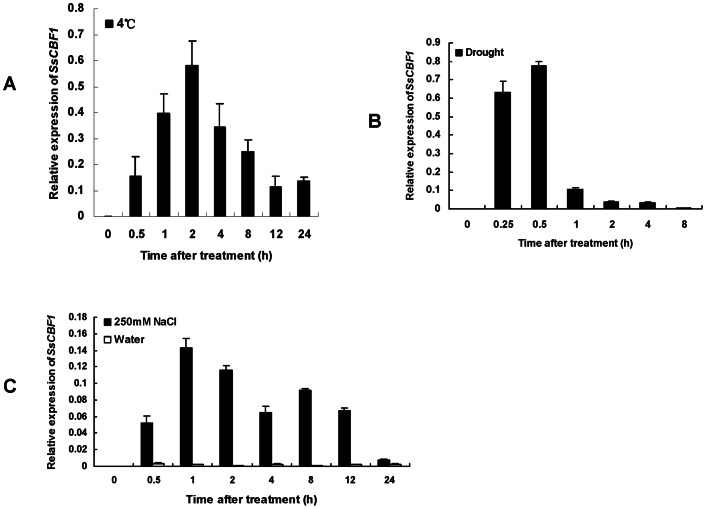
*SsCBF1* transcript levels in response to various abiotic stresses. (**A**) Low-temperature induced expression pattern of *SsCBF1* in *S. lycopersicoides*. *S. lycopersicoides* seedlings grown under standard conditions were transferred to a climate chamber set at 4°C for a 24 time course under constant light. The aerial parts were harvested for RNA extraction and qRT-PCR analysis. Zero time samples were taken prior to treatment. Transcript levels of *SsCBF1* were normalized to the *ACTIN2* expression. (**B**) Drought induced expression pattern of *SsCBF1* in *S. lycopersicoides*. Detached young leaves of *S. lycopersicoides* were placed on a dry filter paper for drought treatment. Samples were collected according to the time course. The *SsCBF1* mRNA levels were analyzed as in (A). (**C**) High-salinity induced expression pattern of *SsCBF1* in *S. lycopersicoides*. Detached young leaves of *S. lycopersicoides* were placed on the filter paper soaked with NaCl solution (250 mM, 0.02% Tween-20) for a high-salinity treatment. Samples were collected at the indicated time points. The *SsCBF1* mRNA levels were analyzed as in (A). Data shown are average and SD of triplicate reactions. Shown are representative data from one biological replicate and three biological replicates were conducted with similar results.

qRT-PCR analysis was used to investigate the expression patterns of the *SsCBF1* gene under various abiotic stresses (Fig. 8). The results showed that *SsCBF1* gene was barely expressed without a treatment (0 h). For cold stress, obvious accumulation of *SsCBF1* transcripts happened after 0.5 h of stress. High level expression of *SsCBF1* was detected during the 1–4 h period of treatment. The *SsCBF1* transcript level decreased but remained significantly higher than that in untreated plants 24 h after the start of the treatment (Fig. 8A). For drought stress, the expression of *SsCBF1* gene was dramatically induced after 0.25 h treatment and reached the peak level at 0.5 h. A sharp decline in the expression of *SsCBF1* was observed at 1 h and the expression was almost undetectable after 8 h of treatment (Fig. 8B). For high-salinity response, a significant accumulation of the *SsCBF1* transcripts was observed after 0.5 h of treatment and the transcripts reached a maximum level at 1 h (Fig. 8C). The expression of *SsCBF1* maintained at a relatively high level until 12 h after the salt stress, and was significantly attenuated at 24 h (Fig. 8C). Previous study demonstrated that *SlCBF1* was only weakly (if at all) responsive to drought and high-salinity treatments [56]. However, our results showed that *SsCBF1* gene could be dramatically induced not only by cold stress but also by drought and high-salinity stresses.

### *35S*:*SsCBF1* transgenic *Arabidopsis* showed increased freezing tolerance and late flowering phenotype

**Figure 9:**
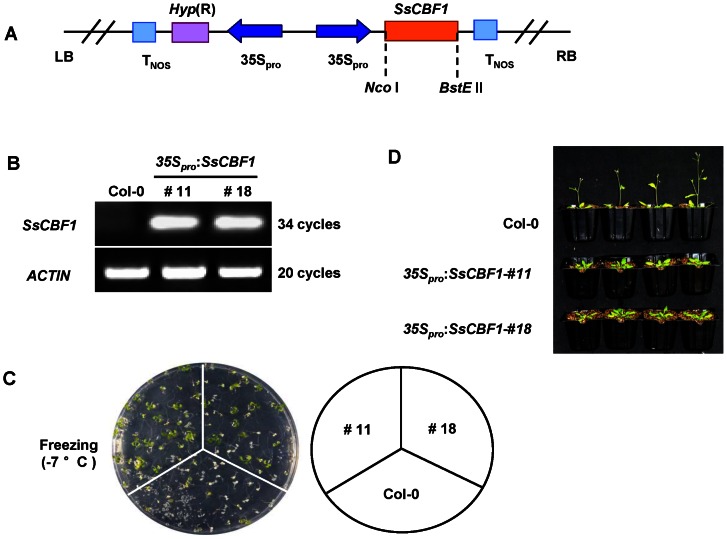
Freezing tolerance and delayed flowering phenotype of *35S:SsCBF1* transgenic *Arabidopsis*. (**A**) Schematic representation of the construct used for *Arabidopsis* transformation of the *SsCBF1* gene. (**B**) Relative expression of *SsCBF1* in Col-0 and two T_3_ generation transgenic lines (#11 and #18). Total RNA was extracted from 10-day-old seedlings, then analyzed by semi-quantitative RT-PCR. *ACTIN7* gene was used as an internal control. (**C**) Comparison of freezing tolerance between Col-0 and two transgenic lines. See Methods for details. The right diagram indicates different genotypes used in the assay. (**D**) Late flowering phenotype of transgenic *Arabidopsis* overexpressing *SsCBF1*. Col-0 and two *SsCBF1* OE lines were grown under the same conditions as described in Methods. Four-week-old plants were photographed.

To further characterize the function of SsCBF1, we generated transgenic *Arabidopsis* plants ectopically overexpressing *SsCBF1* under the control of CaMV35S promoter (Fig. 9A). Two independent T_3_ generation transgenic lines (#11 and #18) were verified by semi-quantitative RT-PCR (Fig. 9B) and were used for the subsequent freezing and high-salinity treatments. The 7-day-old plants grown on MS medium were exposed to –7°C for 8 h of freezing treatment, followed by thawing at 4°C for 12 h in the dark, and then returned to the original growth conditions. The result showed that the survival rates of transgenic plants (#11 and #18) were 45% and 36%, respectively, but only 15% of Col-0 plants survived (Fig. 9C). It was reported that overexpression of *LeCBF1* in *Arabidopsis* caused aberrant growth phenotypes of transgenic plants such as fewer axillary shoots, less seed and delay in flowering [56]. In order to learn whether SsCBF1 plays a role in regulating plant growth, two T_3_ generation transgenic lines (#11 and #18) were monitored on a developmental scale and were compared with similarly monitored Col-0 plants. When grown in parallel, both OE lines showed strong late flowering phenotype. Except for the delay in flowering, no other remarkable differences on growth and development were observed (Fig. 9D).

### Overexpression of *SsCBF1* in *Arabidopsis* enhances plant tolerance to high-salinity

**Figure 10:**
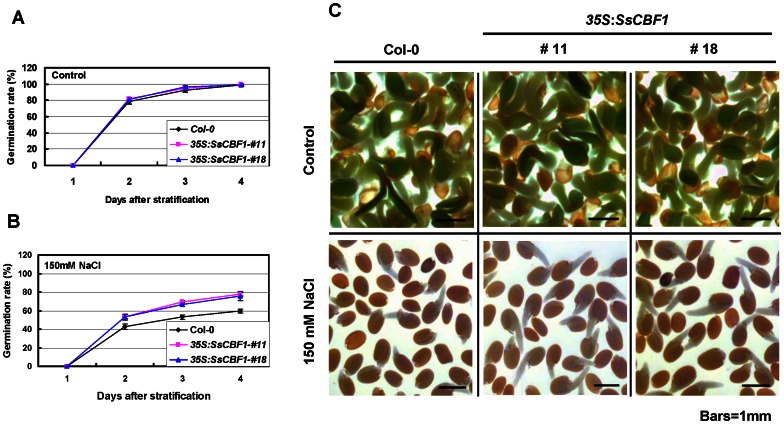
Seed germination of transgenic *Arabidopsis* in response to NaCl. (**A–B**) Seed germination ratio of Col-0 and two OE lines in the absence or presence of NaCl. Seeds from different genotypes were germinated on 1/2 MS agar plates (control, A) or supplemented with 150 mM NaCl (B), respectively. Germination was defined as the obvious sign of radicle tip emergence and scored daily until the indicated day. Data shown are average and SD of four independent experiments (each with 100 seeds for each genotype). (**C**) Increased tolerance of *SsCBF1* transgenic lines to salt stress. Pictures were taken 3 days after stratification.

Under normal growth conditions, there was no noticeable difference in seed germination of two selected transgenic lines (#11 and #18) compared with that of Col-0 (Fig. 10A). However, under high-salinity stress, the germination capability of seeds from the two *SsCBF1*-overexpressing lines was stronger than that observed for Col-0 seeds. For example, in the presence of 150 mM NaCl, less than half of the Col-0 seeds germinated on the second day after stratification, while the germination rates of transgenic seeds were more than 50% (Fig. 10B). Even 3 and 4 days after being transferred to a phytotrone, the seed germination rates of both transgenic lines were still significantly higher than those of Col-0 (Fig. 10B, C).

**Figure 11:**
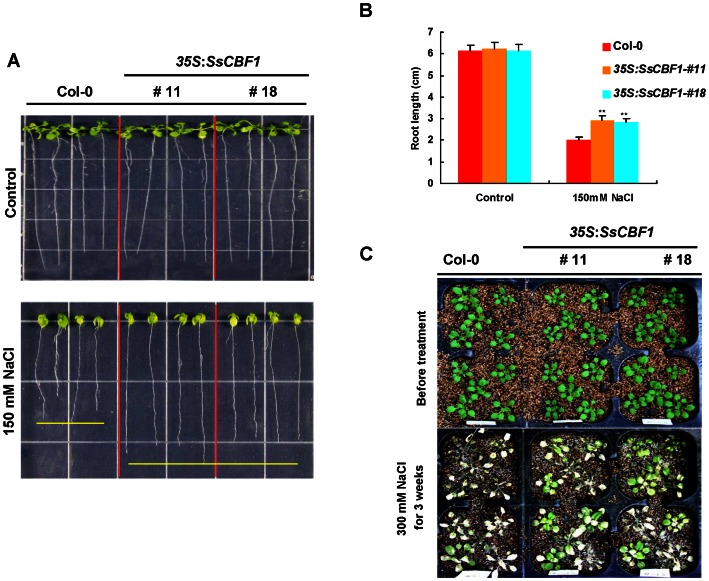
Effects of high salinity on root lengths of Col-0 and *SsCBF1* transgenic lines. (**A**) Representatives of Col-0 and two OE lines treated with 150 mM NaCl. Seeds of each genotype were germinated and grown vertically on MS medium for 4 d and then transferred to fresh MS medium containing 150 mM NaCl. Photographs were taken after 11d of growth. (**B**) Measurements of primary root lengths of plants shown in (A). All values are average and SD (n = 15). Asterisks denote Student's *t* test significance compared with Col-0 plants: **P<0.01. (**C**) High-salinity tolerance of Col-0 and two transgenic adult plants. 3-week-old plants (before treatment, upper panel) were irrigated with 300 mM NaCl solution every 3 d for 3 weeks. Bottom panel was photographed 3 weeks after the onset of irrigation.

To test the high-salinity effects on root length of *SsCBF1* overexpressors, seeds were first germinated and grown vertically on MS agar medium for 4 d, followed by transfer to fresh medium (in the absence or presence of NaCl) for continued vertical growth, after which root length was measured with a ruler and photographed. Under control conditions, there was almost no difference between Col-0 and *SsCBF1* transgenic plants on the growth of primary roots (Fig. 11A, upper panel). The growth of primary roots was significantly inhibited in the Col-0 plants compared with that of *SsCBF1* overexpression lines when grown vertically under the NaCl treatment (Fig. 11A, bottom panel). Statistical analysis of the measurements confirmed that root lengths of the *SsCBF1* transgenic lines were significantly higher (**P≤0.01) than those of Col-0 plants under stress conditions (Fig. 11B). In addition, 3-week-old soil-grown plants were irrigated with 300 mM NaCl for three weeks. The transgenic plants displayed greater salt stress tolerance than Col-0 plants (Fig. 11C).

### RNA-seq analysis of aerial organs in transgenic *Arabidopsis*

In order to investigate the putative members of SsCBF1 regulon in transgenic plants, we compared the gene expression profiles between the *SsCBF1* overexpression line (#11) and Col-0 using RNA-seq approach. Processing of RNA samples on the Illumina HiSeq 2000 system yielded more than 20 million reads, each 100 bp in length, encompassing 2.0 Gb of sequence data for each sample which was then mapped to the reference genome. The statistical analysis identified a total of 338 differentially expressed genes among which 120 were up-regulated and 218 were down-regulated (Table S2) more than two-folds (Q<0.001). Most of these genes are reported to be the components involved in response to stimulus, transducer activity and metabolism. Nineteen stress responsive genes that were up-regulated in plants overexpressing *SsCBF1* are shown in Table 1.

**Table 1:** Up-regulated genes in *35S:SsCBF1* transgenic plants (Q-value <0.001; fold change >2).

ID	Name	Fold	Q-value	Description
AT2G42540.2	COR15a	34.3	0.00E+00	Response to cold, freezing and salt stress
AT1G09350.1	GOLS3	22.97	6.15E-22	Response to cold
AT1G29395.1	Cold regulated 314, inner membrane 1	8.99	3.10E-47	Provide freezing tolerance
AT2G42530.1	COR15b	8.03	3.11E-09	Response to cold
AT1G16850.1	unknown protein	5.74	2.77E-10	Response to salt stress
AT5G04340.1	C2H2(ZAT6)	5.08	7.19E-08	Cold induced zinc finger protein 2
AT3G09940.1	MDAR2(MDAR3)	4.71	7.39E-23	Response to salt stress and water deprivation
AT3G28220.1	TRAF-like family protein	4.08	9.75E-78	Response to salt stress
AT2G47180.1	GOLS1	3.78	6.07E-122	Response to cold and salt stress
AT4G16260.1	Glycosyl hydrolase superfamily protein	3.54	9.05E-14	Response to salt stress
AT3G25780.1	AOC3	3.25	9.05E-14	Response to salt stress
AT5G15970.1	COR6.6 (KIN2)	2.92	1.77E-61	Response to cold, salt stress and water deprivation
AT3G04720.1	PR4	2.70	9.67E-125	Response to salt stress
AT4G27410.3	RD26	2.60	7.92E-07	Response to water deprivation
AT5G52310.1	RD29A (COR78)	2.44	1.34E-18	Response to cold, salt stress and water deprivation
AT3G25770.1	AOC2	2.29	1.69E-15	Response to cold
AT2G04240.1	XERICO	2.26	6.77E-06	Response to salt stress
AT1G52400.1	BGLU18	2.25	3.44E-217	Response to salt stress and water deprivation
AT1G10090.1	ERD4	2.12	7.77E-05	Early-responsive to dehydration stress protein

### Constitutive expression of *SsCBF1* altered cold and salt stress-responsive gene expression

**Figure 12:**
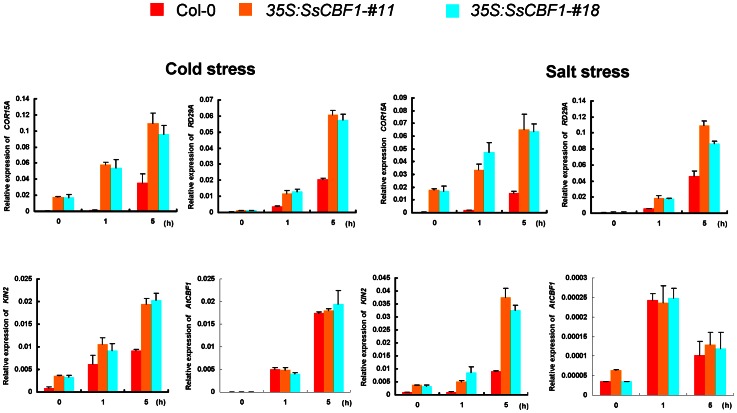
qRT-PCR analysis of stress responsive genes in Col-0 and transgenic *Arabidopsis* seedlings in response to cold or salt stress. The induction of stress-responsive genes (*COR15A*, *RD29A*, *KIN2* and *AtCBF1*) was investigated by qRT-PCR. The experiments were repeated three times with similar results and the data shown are from one representative experiment. Error bars are SD of triplicate reactions.

To further evaluate the role of SsCBF1 in cold and salt stress responses, we chose to monitor the expression patterns of several stress-responsive marker genes in Col-0 and *SsCBF1*-overexpressing lines (#11 and #18) following 4°C exposure or 150 mM NaCl application: *COR15A* [7], *RD29A* [57], *KIN2* [58], [59], and *AtCBF1* [15]. qRT-PCR analysis were performed using gene specific primers. As shown in Figure 12, the transcript levels of these genes could be induced by low-temperature and NaCl stress in the Col-0 and transgenic seedlings with similar kinetics. However, the induction degree of the expression of these genes, except *AtCBF1*, in the transgenic *Arabidopsis* overexpressing *SsCBF1* was substantially higher than that in Col-0 plants after low-temperature and salt stress. In both *SsCBF1* overexpression lines, cold and NaCl-induced expression levels of *AtCBF1* were essentially comparable to those in Col-0 plant. The altered expression patterns of several stress-responsive genes provide a mechanistic explanation of the enhanced freezing and salt tolerance of *SsCBF1* overexpressors.

## Discussion

As sessile organisms, plants have developed protective developmental and physiological strategies to cope with environmental stresses. Low temperature is one of the major limiting factors that could exert negative effect on the yield and distribution of many crops. Therefore, exploring the complex molecular mechanism of cold response pathway has become a crucial subject of agricultural significance in recent years. The CBF transcription factors are the key components mediating the activation of many cold-responsive genes [1], [3]. In our study, a new cold-regulated transcription factor gene was successfully isolated from *S. lycopersicoides* using RACE method and designated as *SsCBF1*. *SsCBF1* encodes a protein of 228 amino acids with a predicted molecular mass of 25.4 kDa which contains nearly all important signature domains of plant CBF proteins such as AP2-DNA binding domain, S repeats and LWNYS motif (Fig. 2A). It is noteworthy that S repeats exist in all *Solanum* CBF1s but not in AtCBF1 (Fig. 2A). Phylogenetic analysis tells us that SsCBF1 is most closely related to SlCBF1 with sequence identity of more than 90% (Fig. 2B). Southern analysis was also used in our research to estimate the number of *SsCBF1*-related genes in *S. lycopersicoides* genome. The result showed that *S. lycopersicoides* contained multiple copies of *CBF*-like genes, constituting a small gene family (Fig. 3).

*Arabidopsis* CBFs could bind to CRT/DRE elements to activate the expression of their target genes. [15]–[17], [60]. Constitutive overexpression of either *LeCBF1* or *AtCBF3* in transgenic tomato plants did not result in an increase in freezing tolerance. It was presumed that this phenomenon could be due to a lack of functional CRT/DRE *cis*-elements in the promoters of target genes [56]. Therefore, it is necessary to test the capability of SsCBF1 to activate the expression of a gene with CRT/DRE elements in its promoter. *Agrobacterium*-mediated transient expression assays showed that SsCBF1 could specifically bind to the CRT elements to activate the expression of downstream reporter genes (Fig. 5, 6). Using Gal4-DBDxSsCBF1 fusion constructs in yeast reporter assays, our functional dissection of the CTAD revealed that the amino acid residues 193–228 (SsCBF1^193–228^) contribute to the activator potential. In addition, replacement of the tryptophan residue with alanine in the LWNYS motif abolishes the full transcriptional activity (Fig. 7).

Previous studies have well established the correlation between CBFs and various abiotic stresses. Three members of *Arabidopsis CBF* gene family (*AtCBF1*, *AtCBF2* and *AtCBF3*) could be rapidly induced by cold stress [9], [61], [62]. The transcript level of *AtCBF4* could be up-regulated by drought but not by low temperature [60]. In this study, evidence from qRT-PCR analysis revealed that the *SsCBF1* transcripts were accumulated quickly and dramatically in response to cold, drought and high-salinity stresses which supports the link between SsCBF1 and these abiotic stresses. *S. lycopersicoide* has been collected from a habitat subjected to chilling, dry and arid areas. It is tempting to conclude that long-term evolution and special survival environment has made SsCBF1 a crucial component mediating the activation of genes responsive to different abiotic stresses and probably other unknown chemical stressors.

Constitutive overexpression of *LeCBF1* gene in transgenic *Arabidopsis* induced the expression of CBF-targeted genes and resulted in an increase in freezing tolerance [56]. In our study, phenotypic analysis was performed using two selected *SsCBF1* overexpression lines (#11 and #18). When the Col-0 and transgenic plants were exposed to −7°C, nearly half of transgenic plants survived, but only 15% of Col-0 plants displayed viability. This result confirmed that *SsCBF1* effectively enhanced the freezing tolerance of transgenic plants. Moreover, ectopic overexpression of *SsCBF1* gene also conferred enhanced salinity tolerance to transgenic *Arabidopsis* (Fig. 10, 11). To further evaluate the role of SsCBF1 in cold and high-salinity responses, the expression patterns of several well-known stress-responsive marker genes (*COR15A*, *RD29A*, *KIN2* and *AtCBF1*) were monitored following cold stress or NaCl exposure (Fig. 12). It was found that these genes were all up-regulated in Col-0 and transgenic plants, but with a higher level in transgenic plants than in Col-0 plants in response to cold and salt stress. It is also noteworthy that the expression patterns of *AtCBF1* were similar in Col-0 and OE lines after low-temperature and salt treatment. Therefore, the stronger induction of these stress-related genes in OE lines might be largely due to the accumulation of SsCBF1 but not the endogenous regulators. Although the expression of only a limited number of salt-stress markers was substantially higher in *SsCBF1* overexpression lines than in Col-0 plants after high-salinity treatment, it is still reasonable to expect a salt-tolerant phenotype. The increased salt tolerance of transgenic *Arabidopsis* might also be due to the expression changes of other uncharacterized genes which are involved in the osmotic stress regulatory network of the plant.

Plant flowering is under the control of both environmental stimuli and endogenous cues. Several pathways affecting flowering such as photoperiod, vernalization and GA pathways have been extensively reviewed [63]–[65]. In our study, bolting and flowering of transgenic plants were delayed by several days as compared with Col-0 plants (Fig. 9D) and no other differences in growth and development could be observed. It is intriguing that *AtMAF5* (AT5G65080), a negative regulator of flowering time, was up-regulated more than 25-fold (Table S2). The up-regulation of *AtMAF5* is very likely to contribute to the delayed flowering of transgenic *Arabidopsis*.

The expression of 8000 *Arabidopsis* genes was surveyed by transcriptome analysis [20] and it was reported that approximately 4% (306) genes were up- or down- regulated at least threefold in response to low temperature. However, at least 28% of those genes were not regulated by CBF transcription factors, and 12% genes were considered to be certain members of CBF regulon. Therefore, many previous studies concluded that low temperature not only activated the CBF regulated pathway, but also activated other multiple low temperature regulatory pathways [22], [66], [67]. To better understand the function of *SsCBF1*, we conducted RNA-seq experiments using the Illumina Hiseq 2000 system because of its superiority over the traditional microarray methods [47], [68], [69]. Gene expression comparison using our RNA-seq data confers the identification of a robust set of cold-responsive genes that could be used to advance us toward deciphering the cold response regulatory networks. A total of 120 genes (*P*≤0.001) were up-regulated in *SsCBF1* transgenic *Arabidopsis*. Gene Ontology (GO) and Pathway analysis divided the up-regulated genes into five groups: environmental adaption, infectious diseases, transport and catabolism, signal transduction and metabolism (Table S3). The complete gene expression profiling of our materials is provided in Table S4.

In conclusion, we characterized *SsCBF1* as a cold, drought and high-salinity responsive gene in the nightshade plant *S. lycopersicoides*. *Arabidopsis* overexpressing *SsCBF1* showed late flowering trait and increased freezing and high-salinity tolerance. Although the functional exploration of SsCBF1 is far from accomplished, the data we present here is of value for genetic modification of many economically important crops.

## Supporting Information

Table S1 **List of the primers used in this study.**
(XLS)

Table S2 **List of the differentially regulated genes in** ***SsCBF1*** **OE plants.**
(XLS)

Table S3 **Pathways and Gene Ontology (GO) analysis of the RNA-seq data.**
(XLS)

Table S4 **Transcription profiling of** ***Arabidopsis*** **plants overexpressing** ***SsCBF1*** **.**
(XLS)
